# Determination of parecoxib and valdecoxib in rat plasma by UPLC-MS/MS and its application to pharmacokinetics studies

**DOI:** 10.1186/s40360-020-00406-5

**Published:** 2020-04-07

**Authors:** Mengchun Chen, Wei Sun, Zhe Wang, Chengke Huang, Guoxin Hu, Yijie Chen, Ledan Wang

**Affiliations:** 1 Department of Pharmacy, The Second Affiliated Hospital, and Yuying Children’s Hospital of Wenzhou Medical University, Wenzhou, 325000 Zhejiang China; 2 School of Pharmacy, Wenzhou Medical University, Wenzhou, 325000 Zhejiang China; 3 Department of Obstetrics and Gynecology, The Second Affiliated Hospital and Yuying Children’s Hospital of Wenzhou Medical University, No. 109, Xueyuan West Road, Lucheng District, Wenzhou, Zhejiang China

**Keywords:** Parecoxib, Valdecoxib, UPLC–MS/MS, Rat plasma, Pharmacokinetics

## Abstract

**Background:**

The present study aimed to develop and validate a rapid, selective, and reproducible ultra-performance liquid chromatography-tandem mass spectrometry separation method for the simultaneous determination of the levels of parecoxib and its main metabolite valdecoxib in rat plasma. Moreover, this method was applied to investigate the pharmacokinetics of parecoxib and valdecoxib in rats.

**Methods:**

Following the addition of celecoxib as an internal standard, one-step protein precipitation by acetonitrile was used for sample preparation. The effective chromatographic separation was carried out using an ACQUITY UPLC®BEH C18 reversed phase column (2.1 mm × 50 mm, 1.7 μm particle size) with acetonitrile and water (containing 0.1% formic acid) as the mobile phase. The procedure was performed in less than 3 min with a gradient elution pumped at a flow rate of 0.4 ml/min. The electrospray ionization source was applied and operated in the positive ion mode and multiple reaction monitoring mode was used for quantification using the following: target fragment ions: m/z 371 → 234 for parecoxib, m/z 315 → 132 for valdecoxib and m/z 382 → 362 for celecoxib.

**Results:**

The method validation demonstrated optimal linearity over the range of 50–10,000 ng/ml (r^2^ ≥ 0.9996) and 2.5–500 ng/ml (r^2^ ≥ 0.9991) for parecoxib and valdecoxib in rat plasma, respectively.

**Conclusions:**

The present study demonstrated a simple, sensitive and applicable method for the quantification of parecoxib and its main pharmacologically active metabolite valdecoxib following sublingual vein administration of 5 mg/kg parecoxib in rats.

## Background

Parecoxib (PCX) is an injectable prodrug of valdecoxib (VCX) that has been widely applied as a second-generation nonsteroidal cyclooxygenase 2 (COX-2) selective inhibitor. This compound was approved in the clinic from 2002 for short-term perioperative pain management [1]. A specific dose of PCX was used for the control of acute pain and the onset of analgesia was set at the first 7–14 min and reached its peak effect within 2 h. In general, the duration of analgesia after a single dose is both dose- and clinical pain model-dependent and approximately ranges from a time period of 6 to higher than 24 h [2]. Clinical trials have indicated that PCX is effective in relieving postoperative pain, including oral surgery, orthopedic surgery and abdominal hysterectomy pain. PCX exhibited negligible adverse effects on cyclooxygenase-1 (COX-1) inhibition which this inhibitory effect could cause a series of severe complications such as gastroduodenal ulceration, bleeding and platelet function compromise [3]. These characteristics allow PCX treatment of a wider group of patients [4]. However, certain studies have shown that PCX and VCX increase cardiovascular risk in post-surgical patients at a dose-dependent manner [5–8]. PCX can be rapidly converted to the active COX-2-specific compound VCX and to propionic acid in the plasma, liver and other tissues [9, 10]. The majority of the metabolites are excreted by the urine [9, 10]. Previous studies have shown that the cytochrome P450 3A4 and 2C9 enzymes are mainly involved in PCX metabolism [11–13]. Therefore, the determination of PCX and its major metabolite is required to precisely detect its concentration levels in the blood circulation when used with cytochrome P450 3A4 and 2C9 inducers or inhibitors.

Valdecoxib (VCX) is the metabolite of parecoxib (PCX) and contains a sulfonamide group, which is replaced by a sulfonyl propanamide in PCX [14]. Following systemic delivery, the fate of VCX is determined as follows: This compound is highly bound to plasma proteins (98%) and subsequently metabolized primarily by cytochrome P450 3A4 (CYP3A4) and by cytochrome P450 2C9 (CYP2C9) as a secondary metabolic route. The metabolism of VCX yields a variety of metabolites that are finally excreted in the urine [15–17]. A hydroxylated metabolite of VCX (via the CYP-450 pathway) has been identified in human plasma that is demonstrated as another active COX-2 inhibitor albeit with weaker inhibitory effect than VCX [18]. However, approximately 10% of VCX in the circulation is metabolized to hydroxylated VCX that exerts a slight clinical effect compared with that of its parent molecule VCX, although both compounds exhibit similar pharmacokinetic characteristics. Therefore, the detection of the concentration of the hydroxylated metabolite of valdecoxib is not necessary [19]. Since VCX is a substrate for hepatic CYP2C9 and CYP3A4 enzymes and both PCX and VCX are inhibitors of CYP2C9 and CYP2C19, PCX and VCX may interact with other similarly in structure drugs. Therefore, the concentration levels of PCX and VCX would be changed as the activity of hepatic enzymes be induced, or suppressed. Hence, the development of a rapid and accurate separation method for the simultaneous determination of PCX and its metabolite VCX in plasma is mandatory.

To the best of our knowledge, the reports on the simultaneous detection and quantification of PCX and its primary active metabolite VCX in biological matrices by ultra-performance liquid chromatography-tandem mass spectrometry (UPLC-MS/MS) [20] or LC-MS/MS are rare [19]. Although the aforementioned two methods are effective, the preparation process is complicated. The chromatographic assays must be combined with a liquid-liquid extraction strategy followed by complete evaporation of organic solvents [20]. In addition, the two methods require a lengthy analysis time of 7.5 min for each sample that is considered as time-consuming [19]. In this regard, the present study aimed to develop and validate a simple and convenient UPLC-MS/MS method to simultaneously quantify PCX and VCX levels in plasma samples. A rat model was selected in the present study to examine PCX metabolism in vivo.

In the current study, we established an UPLC-MS/MS method with selectivity and reproducible for determination of PCX and its metabolite VCX simultaneously in rat plasma samples. This method displayed high preciseness and accuracy in analyzing quality control samples regardless of how to process them including either freeze-thaw cycles, dilution, or storage for a long time. Following the availability of the developed method, the pharmacokinetics both of PCX and VCX in rat plasma were subsequently investigated after administration of a given dose of PCX.

## Methods

### Chemicals and reagents

Parecoxib, valdecoxib, and celecoxib, all of which with purity > 98.0% were obtained from Sigma-Aldrich (St. Louis, MO, USA). LC-MS grade acetonitrile and formic acid (98% purity) were procured from Merck (Darmstadt, Germany) and Sigma-Aldrich (Munich, Germany), respectively. Other organic solvents born with HPLC grade were purchased from Merck (Darmstadt, Germany). It is worth mentioning that the rest of the reagents employed throughout this study were of analytical pure without further purification, include the ultra-pure water, which was yielded by a Millipore Milli-Q purification system (Billerica, MA, USA).

### Instrumentation and conditions

ACQUITY I-Class UPLC (Waters Corp., Milford, MA, USA) was consist of a Quaternary Solvent Manager (QSM), a Sample Manager with Flow-Through Needle (SM-FTN), and additionally integrated with a XEVO TQD triple quadrupole mass spectrometer (Waters Corp., Milford, MA, USA). Look further on the spectrometer, there was an Electrospray ionization (ESI) source equipped with that was controlled by inside Masslynx 4.1 software (Waters Corp., Milford, MA, USA).

Samples were analyzed by an ACQUITY I-Class UPLC using an ACQUITY UPLC®BEH C18 column (2.1 mm × 50 mm, 1.7 μm particle size, Waters, USA) that kept at 40 °C, and a mobile phase composed of acetonitrile-water (containing 0.1% formic acid) flowed in an inline 0.2 μm stainless steel frit filter (Waters Corp., Milford, USA). The autosampler were at a constant temperature of 4 °C. Varied ratios of acetonitrile (A) and water containing 0.1% formic acid (B), including 0–0.5 min (60% A), 0.5–1.5 min (60–95% A), and 1.5–2 min (95–60% A) were worked as a gradient elution procedure to achieve chromatographic separation. During the whole process, kept each sample input volume at 2 μl and the mobile phase flowed at a rate of 0.4 ml/min, and all workflow for each sample might cost about 3 min.

Electrospray ionization (ESI) source in XEVO TQD triple quadrupole mass spectrometer was set up at positive ion mode to perform Mass spectrometric analysis. Nitrogen applied in the system was both as a desolvation gas and cone gas with a flow rate at 600 L/h and 50 L/h, respectively. Following the basic settings, the selected ionization parameters were below: 4 kV of capillary voltage, 150 °C of source temperature, and 500 °C of desolvation temperature. Also, a list that contained a series of multiple reaction monitoring (MRM) fragmentation transitions and MS parameters were displayed in Table 1.

**Table 1:** MS parameters for parecoxib, valdecoxib, and celecoxib (IS)

Analytes	Parent [M + H] + (m/z)	daughter(m/z)	Dwell(s)	cone(V)	collision (eV)
**Parecoxib**	371	234	0.108	40	20
**Valdecoxib**	315	132	0.108	40	20
**Celecoxib**	382	362	0.108	60	30

### Calibration standards and quality control (QC) samples

PCX, VCX, and internal standard (IS) were made individually, all which were dissolved in methanol at an identical concentration of 1 mg/ml as stock solutions and then stored at 4 °C. All samples were adjusted to room temperature prior to use, and the resultant stock solutions were further diluted by untreated rat plasma to make different work concentrations.

The calibration curves were plotted using given concentrations including 50, 100, 500, 1000, 5000, 10,000 ng/ml for PCX, and 2.5, 5, 25, 50, 250, 500 ng/ml for VCX. Also, the QC samples with planned three concentrations of 100, 800, 8000 ng/ml for PCX, and other three concentrations of 5, 40, 400 ng/ml for VCX were made and aliquoted to 100 μl per tube and then stored at − 20 °C before use.

### Sample preparation

Frozen samples were thawed and recovered completely to room temperature in advance for further analysis. 20 μl of the IS at a concentration of 1 μg/ml was mixed with 100 μl of plasma samples. After that, 200 μl acetonitrile was added to the as-prepared IS-plasma mixture for protein precipitation. Following the mixing for 2 min, the resultant solutions were centrifuged at 13,000 r/min for 10 min at 4 °C, and the resulting 100 μl supernatant was collected and diluted with an equal volume of ultra-purified water. Upon this moment, PCX, VCX, and IS contents in samples were ready to be analyzed by the UPLC-MS/MS system.

### Method validation

#### Specificity and matrix effect

The potential interference existing in samples was determined as a specificity of methodology. In this study, blank plasma samples, PCX, VCX and IS mixed with the blank plasma, the plasm samples collected from the rat that intravenously injected with 5 mg/kg PCX, were analyzed to compare with each other, and all of which confirmed the absence of potential endogenous interference in rat blood samples.

The matrix effect was defined by the ratio that divided the peak area of to-test samples (blank plasma mixed with varying contents of QC samples including 100, 800, 8000 ng/ml for PCX, and 5, 40, 400 ng/ml for VCX; *n* = 6) by the peak area of neat standard solutions at the identical concentrations. Also, the matrix effect of IS (200 ng/ml, *n* = 6) was tested using the same protocol. The acceptable relative standard deviations (RSD) bias should locate within ±15%.

#### Calibration curve and LLOQ

The linear regression analysis was performed upon the peak area ratios of plasma samples to IS concentrations, which were fitted in the range of 50–10,000 ng/ml for PCX and 2.5–500 ng/ml for VCX. The weighting factor of the reciprocal of the concentration (1/x) was used to fit the standard curves. The lower limit of quantification (LLOQ) described the detectable lowest level regarding calibration curves, which meets two rules, including the RSD within 20% of the established range, and the signal-to-noise ratio is greater than 10 at least.

#### Precision, accuracy, and recovery

The precision was tested by the first day and later two consecutive days measurements on QC and IS samples with given concentrations. The accuracy was calculated through a formula that the concentration of samples was divided by the predicted concentration theoretically. The acceptable value of the relative error (RE) was less than 15%, and the RSD was within ±15%.

The extraction recoveries on both QC and IS samples were calculated by comparing the peak area ratio of the extracted samples to the peak area ratio in the pure standard extract. The acceptable extraction recovery is higher than 50% for all samples.

#### Stability

The stability of the samples (including QC and IS) in rat plasma was determined by placing samples (*n* = 6) in three settings. Of those settings, the short-term stability was determined via leaving the samples at room temperature for 24 h. For long-term stability evaluation, the samples went through 3 weeks of storage at − 20 °C before measurement. The stability of samples after freeze-thaw treatment was assessed by performing freeze/thaw cycles on each sample three times prior to analysis. The fresh samples were prepared as a negative control. The acceptable bias was considered great stability when it was within ±15%.

### Pharmacokinetic study

The average weight range of 200–220 g male Sprague Dawley (SD) was obtained from the laboratory animal center of the Wenzhou Medical University (Wenzhou, China, License No. SCXK [ZJ] 2005–0019). Rats stayed in cages and freely got food and water under a stable temperature range of 24–26 °C and a controllable 12 h light/dark cycle apparatus. Animal protocols were approved by the Institutional animal experimentation Committee of the Wenzhou Medical University. A total of 12 SD rats were separated into two isolated groups, including the experimental group (*n* = 6) and the control group (n = 6). Fasting was carried out for 12 h before assays, and only water was freely accessible. The rats were treated with 5 mg/ml PCX intravenously, which was equivalent to 56 mg for an individual of 70 kg human body weight (regular range in the clinic is from 40 to 80 mg). 0.3 ml of blood samples were collected and stabilized in heparinized tubes at each given time point (0, 0.083, 0.167, 0.25, 0.5, 0.75, 1, 2, 3, 4, 6, 8, 10, 12 and 24 h). The plasma samples were separated through centrifugation at 3000 r/min for 10 min and carefully collected the supernatant and stored at − 20 °C before analysis. All pharmacokinetic data were analyzed by the DAS (Drug and Statistics) software. All rats were sacrificed by CO_2_ inhalation.

## Results

### UPLC-MS/MS conditions

**Fig. 1:**
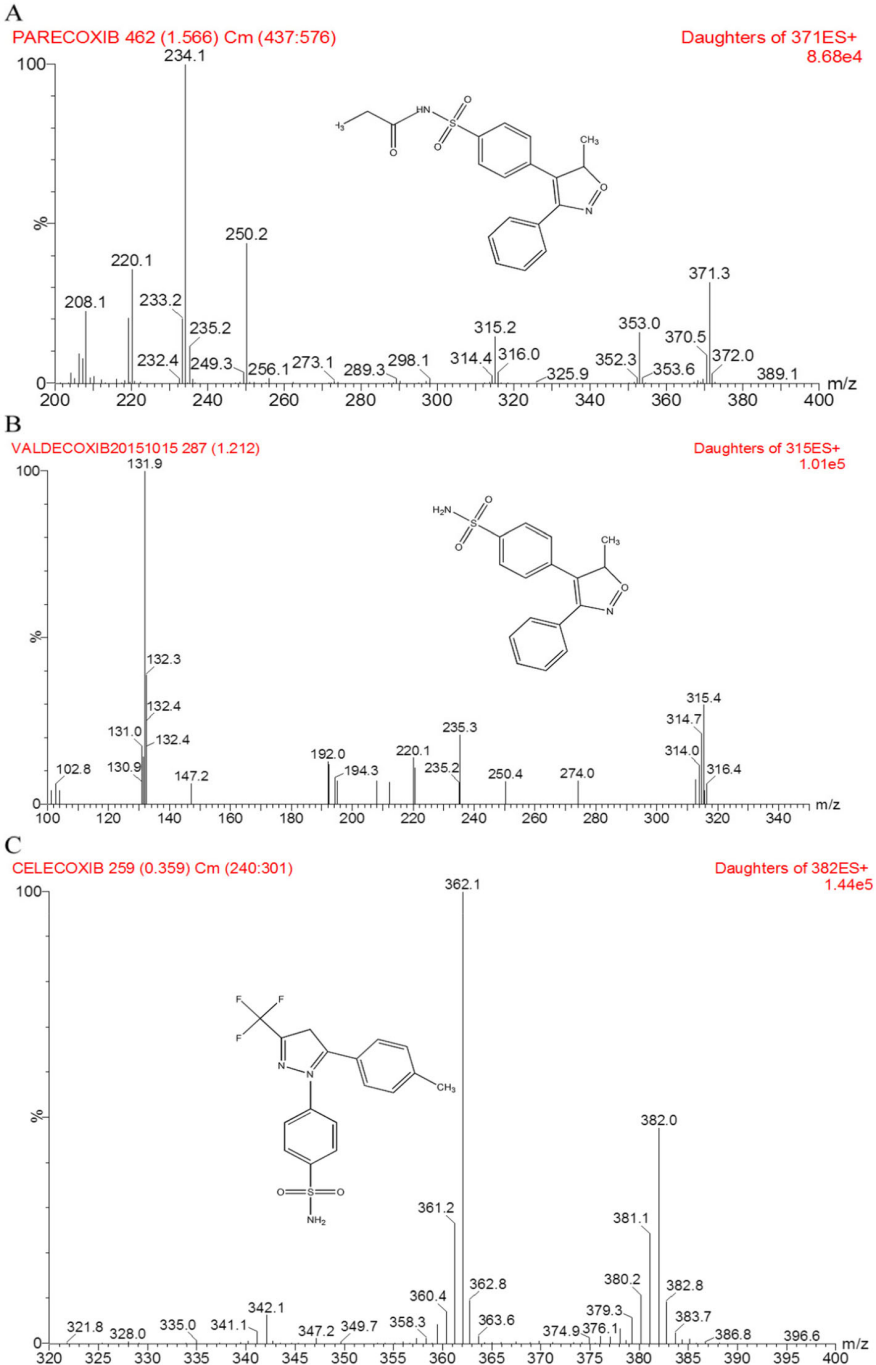
The chemical structures and daughter scan ion spectra of two analytes and IS in the present study: **a** PCX; **b** VCX; **c** celecoxib (IS)

A sensitive and specific UPLC-MS/MS method was established to quantify the blood level of PCX and VCX. The celecoxib was picked as an internal standard, and the purified sample was obtained using one-step protein precipitation with acetonitrile. The effective chromatographic analysis was carried out using an ACQUITY UPLC®BEH C18 reversed-phase column (2.1 mm × 50 mm, 1.7 μm particle size) with a mobile phase of acetonitrile and water (containing 0.1% formic acid) at a flow rate of 0.4 ml/min. As a result, the retention times for PCX, VCX, and IS were about 1.11 min, 0.76 min, and 1.83 min, respectively. The positive ion mode of electrospray ionization source was performed and along with the quantification via the target fragment ions in multiple reaction monitoring modes. The following ions were used: m/z 371 → 234 for parecoxib, m/z 315 → 132 for valdecoxib, and m/z 382 → 362 for celecoxib. The product-scan spectra of the molecular ions of the PCX, VCX, and IS following direct injection in 1: 1 volume ratio of acetonitrile to water are shown in Fig. 1.

### Method development and validation

#### Specificity and matrix effect

**Fig. 2:**
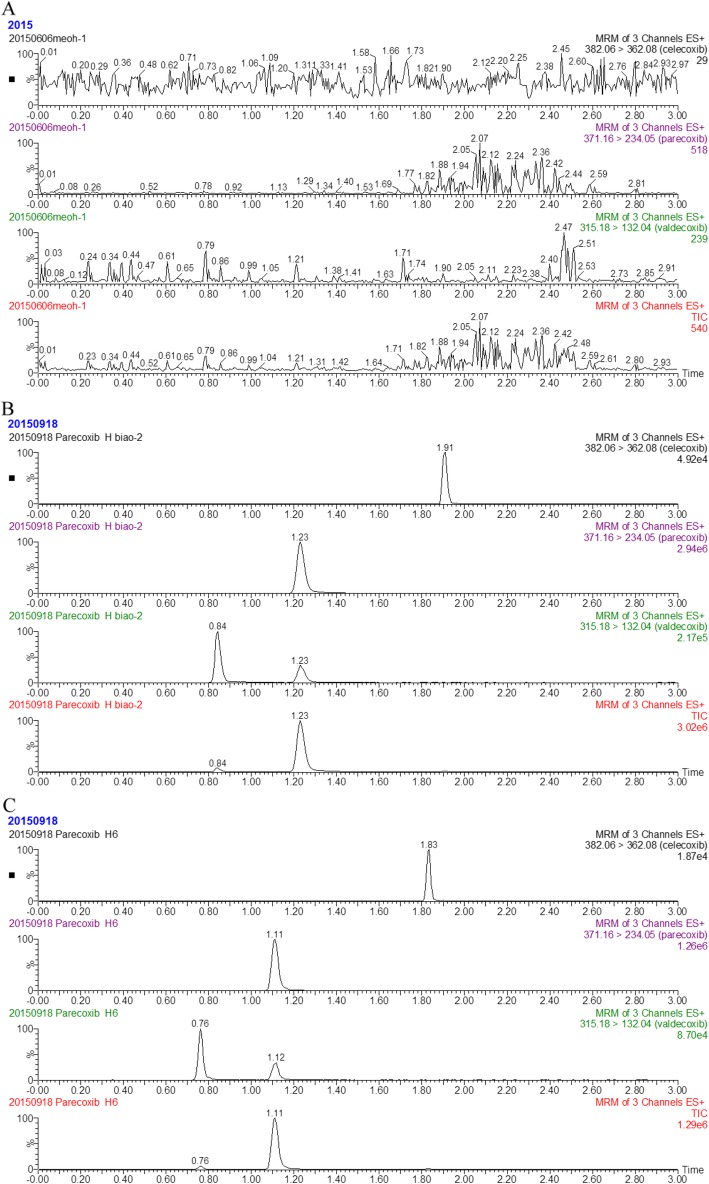
Representative chromatograms of PCX, VCX and IS in rats plasma samples. **a** a blank plasma sample; **b** a blank plasma sample spiked with PCX, VCX and IS; **c** a plasma sample from a rat after sublingual vein administration of 5 mg/kg parecoxib

To identify the specificity of method, three experimental groups were prepared and tested by UPLC-MS/MS. As shown in Fig. 2, the representative chromatographs were compared with each other from those groups, including a blank plasma (Fig. 2a), a blank plasma mixed with the known concentration of PCX, VCX and IS (Fig. 2b) and a plasma sample collected from a rat that treated with 5 mg/kg PCX intravenously (Fig. 2c). Reflection from the above results indicated that there was negligible endogenous interference from the plasm sample spiked with PCX, VCX, and IS or the sample directly harvested from PCX treated rat.

On the other hand, the matrix effect of QC and IS samples were investigated, and subsequent results presented that the QC sample exhibited the range of 94.9 to 109.9% at the three-set concentrations (*n* = 6), and IS within 101.1 ± 1.6% (*n* = 6), which suggesting the matrix effect is negligible.

#### Calibration curve and LLOQ

To fit the peak area ratio of the plasma sample to IS, linear regression analysis was employed. The given ranges of 50–10,000 ng/ml for PCX and of 2.5–500 ng/ml for VCX were tested. The weighted (1/x^2^) least-square regression function was applied to calculate the coefficient, in which the equations were below: Y = 0.122307*X + 5.64622 (r^2^ = 0.9996) for PCX and Y = 0.115791*X + 0.0719761 (r^2^ = 0.9991) for VCX, where Y and X represented the peak area ratios of the analytes to IS and the concentration of the analytes in rat plasma (ng/ml), respectively. The detection was set the signal-to-noise value greater than 10, and that also was set as the LLOQ concentration levels for the analytes in rat plasma. In this study, the LLOQ of PCX was 50 ng/ml, and the resultant precision and accuracy for LLOQ were 12.9 and 14.2%, respectively. Also, the LLOQ of VCX was 2.5 ng/ml, with the precision and accuracy of LLOQ at 11.7 and 14.8%, respectively.

#### Precision, accuracy, and recovery

The precision and accuracy based on intra- and inter-day were determined by the first day and later two consecutive days measurements on QC and IS samples with given concentrations. Upon the analysis, intra-day precisions were 10.5 and 9.5% or less, and the inter-day precisions were 13.9 and 7.5% or less for PCX and VCX, respectively. The intra- and inter-day precisions for IS were 3.8 and 4.8%, respectively. The accuracy and precision data for all analytes were listed in Table 2. All data met the FDA criteria for biological samples analysis. Consequently, results were all within the acceptable range that conferred the current method with high preciseness and accuracy.

**Table 2:** The Intra- and Inter-day precision and accuracy (*n* = 6), extraction recovery (*n* = 6) for parecoxib, valdecoxib and celecoxib (IS) in rat plasma

Compound	Concentration (ng/mL)	Intra-day		Inter-day		Recovery	
		Precision (RSD%)	Accuracy (RE%)	Precision (RSD%)	Accuracy (RE%)	Mean + SD (%)	RSD (%)
	100	8.3	4.3	11.5	10.5	87.0 ± 1.8	2
**Parecoxib**	800	10.5	−3.2	13.9	6.6	79.0 ± 5.0	6.4
	8000	5.6	6	11.4	−7	87.1 ± 0.4	0.5
	5	9.5	5.6	14	−5.6	114.6 ± 8.4	7.3
**Valdecoxib**	40	7.3	1.2	7.1	14.8	79.3 ± 1.1	1.4
	400	4.9	2.9	7.5	6.4	70.0 ± 1.4	2.0
**Celecoxib**	200	3.8	7.3	4.8	8	79.5 ± 2.0	2.6

Also, the extraction recovery of all analytes was summarized in Table 2. Briefly, QC samples had a range of 94.6 to 105.5% at given three concentrations, and the IS was 90.4%. Therefore, the current method was considered a high recovery efficacy.

#### Stability

Following the three designed experimental settings, including short- and long-term and freeze-thaw cycles, all analytes showed stable due to the concentration bias within ±15% of nominal value. Therefore, the reliable pharmacokinetic results were able to achieve via this method. All relevant data has shown in Table 3.

**Table 3:** Stability of parecoxib, valdecoxib and celecoxib (IS) under various conditions (*n* = 6)

Compound	Concentration (ng/mL)	Short-term (room temperature, 24 h)		Long-term (−20 °C, 3 weeks)		Freeze/thaw (−20 °C to room temperature)	
		RSD(%)	RE(%)	RSD(%)	RE(%)	RSD(%)	RE(%)
	100	12.2	13.0	12.9	14.2	12.8	14.9
**Parecoxib**	800	5.2	10.2	11.1	12.7	8.4	14.2
	8000	6.5	−10.5	6.1	14.3	13.4	−7.9
	5	10.6	−6.7	13.2	−1.4	14.9	5.2
**Valdecoxib**	40	7.6	12	7.5	13.2	8.4	14.2
	400	5.9	3.2	7.7	3.2	7	9.5
**Celecoxib**	200	4.2	7.5	5.2	−1.3	5.2	5.8

#### Application of the method in a pharmacokinetic study

**Fig. 3:**
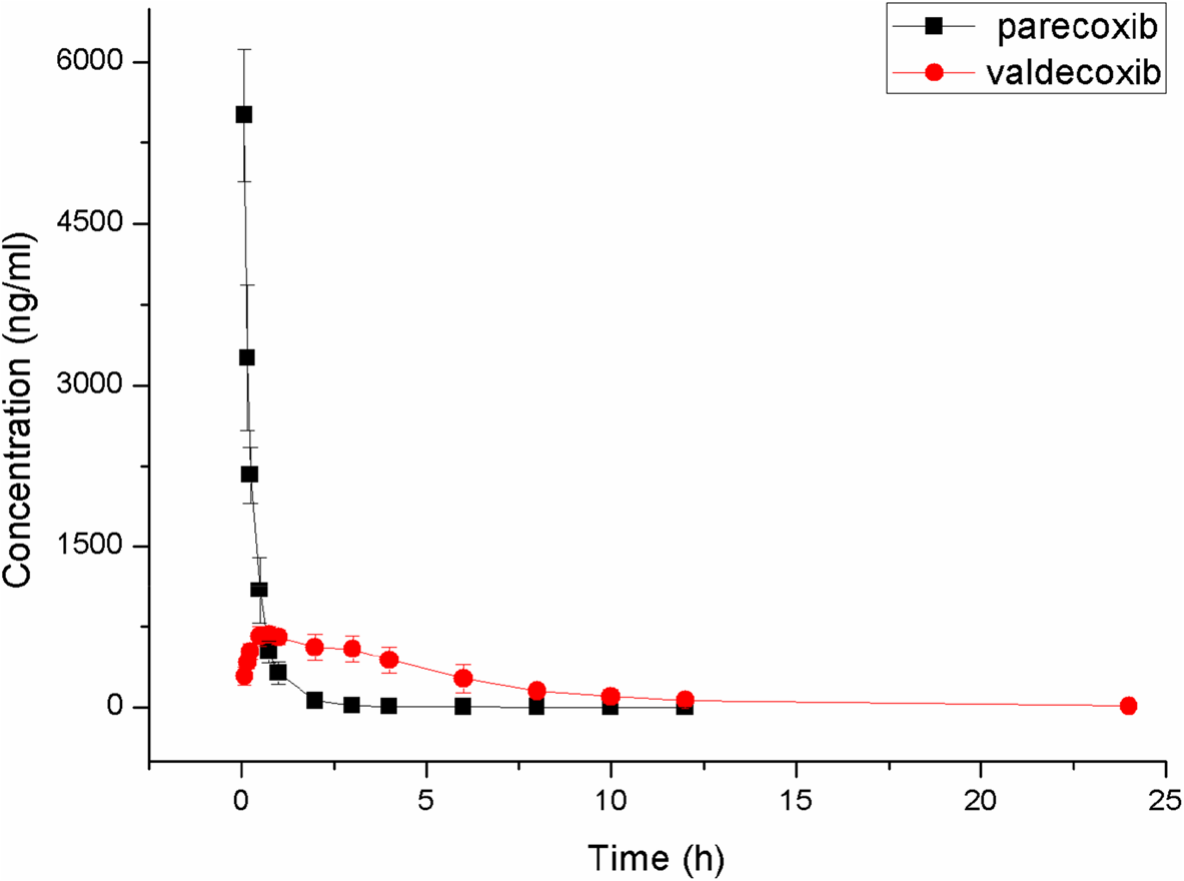
Plasma concentration versus time curves of PCX and VCX for six rats after sublingual vein administration of 5 mg/kg parecoxib

In the clinic, the recommended dose of PCX was 40 mg per patient, i.m. or i.v., and the total daily dose was not more than 80 mg. In the present study, a dose of 5 mg/kg PCX was injected to rats by i.v., which was equivalent to 56 mg for an individual of 70 kg body weight (range of 40–80 mg). The UPLC-MS/MS has effectively monitored the pharmacokinetic changes after administration of 5 mg/ml PCX. Further, the pharmacokinetic parameters were figured out by using the DAS 3.0 software. The two-compartment model was used to analyze the critical pharmacokinetic parameters displayed in Table 4. Also, the described concentration-time curve of PCX in plasma has shown in Fig. 3.

**Table 4:** Pharmacokinetics parameters of the parecoxib and valdecoxib after sublingual vein administration of 5 mg/kg PCX in rat (*n* = 6)

Parameter	Parecoxib	Valdecoxib
AUC(0-t)(ug/L*h)	2106.8 ± 282.3	4186.1 ± 1593.0
AUC(0-∞)(ug/L*h)	2108.0 ± 282.6	4371.7 ± 1526.3
**MRT(0-t)(h)**	0.4 ± 0.1	4.1 ± 1.0
**MRT(0-∞)(h)**	0.4 ± 0.1	4.8 ± 1.1
**t**_**1/2**_**(h)**	1.4 ± 0.5	3.1 ± 1.1
**Tmax(h)**	0.1 ± 0.0	0.8 ± 0.2
**CL(L/h/kg)**	2.4 ± 0.3	1.2 ± 0.4
**Cmax (ug/L)**	5066.4 ± 1207.9	700.6 ± 92.6

## Discussion

Sample preparation is a crucial step that determines the fate of biological sample analysis. Blood samples contain a substantial quantity of endogenous factors that may interfere with the quantification of the analytes. For this purpose, a viable extraction protocol should maximize drug recovery with minimum noise. Frequently-used plasma extraction methods mainly include liquid-liquid extraction (LLE), protein precipitation, and solid-phase extraction (SPE) [21]. LLE is a popular method of sample extraction that has been used in our preliminary experiments. In these studies, we failed to obtain a suitable recovery rate. In the advanced LLE method, additional chemicals, such as 0.1% formic acid, ethyl acetate: diethyl ether (3:1, v/v) and 50% methanol in water can be used to prepare plasma samples, which makes the sampling process very tedious, as demonstrated previously [20]. The SPE method can achieve a high recovery rate and excellent precision. However, it includes a high cost, complicated steps, and involves a variety of organic solvent extraction methods that impede its wide applications. Based on this evidence, the protein precipitation method was applied, which is simple, convenient, fast, and frequently used. The experimental conditions were optimized by changing the different organic solvents, such as acetonitrile, ethanol, methanol, and perchloric acid in order to achieve optimal extraction recovery. To this end, the one-step protein precipitation method was employed in the current study.

The traditional method often adopts high-performance liquid chromatography (HPLC) for the determination of PCX and VCX [22]. However, HPLC requires long-time sample running and exhibits low sensitivity. Therefore, a more sensitive, specific, and straightforward method of UPLC-MS/MS was applied in the current study in order to determine the levels of both PCX and VCX with increased precision and sensitivity. Chromatographic condition settings are a prerequisite to acquiring reliable results, and therefore the optimization of the conditions is required. The mobile phase was optimized by evaluating the percentage of methanol and acetonitrile individually, and the acetonitrile was subsequently selected as the organic phase due to the lowest background noise. In addition, the mobile phase was supplemented with 0.1% (v/v) formic acid to obtain symmetrical peak shapes and to improve ionization efficiency [23]. Alternatively, previous studies used 0.5 mM of either ammonium formate [19] or ammonium acetate [20] instead of formic acid, and the analysis resulted in distinct peak shape. However, both ammonium formate and ammonium acetate can inhibit ionization, and therefore formic acid was used.

An ACQUITY UPLC®BEH C18 column attached with an inline filter was used in this study. The method achieved a rapid, efficient analysis for analytes. Separated peaks for PCX, VCX, and IS were evident with optimal sensitivity using gradient elution under a mobile phase consisting of fixed acetonitrile to water with 0.1% formic acid in aquatic phase. The entire running time was less than 3 min and satisfied further high-throughput clinical analysis.

PCX, VCX, and IS received hydrogen ions readily to form positive ions, while nitrogen-containing compounds, such as R-NH_3_^+^ or R2-NH_2_^+^, were introduced. Moreover, tested with higher signal intensity for PCX, VCX, and IS, the positive ion model in mass spectrometer was selected in the present study. After optimization of MS parameters (containing desolvation gas temperature, capillary voltage, collision energy, source temperature, nitrogen flow rate, and so on), all which lead to enhanced sensitivity for each analyte. Besides customized MS parameters, others were as regularly followed in the instrumental direction.

The IS plays a pivotal role in establishing a method. Celecoxib has a similar molecular structure to parecoxib or valdecoxib and can be used as an optimal IS due to its stability, absence of matrix effects, and reproducible extraction features.

The exposure levels of VCX (AUC, Area under the plasma concentration-time curve, and C_max_, Peak plasma concentration) were almost the same following i.v. or i.m. injection and the concentration levels of PCX were the same (AUC), whereas the average C_max_ of PCX following i.m. was lower than that noted by i.v. administration, which may be due to the slow extravascular absorption of drugs caused by i.m. injection. Since the plasma concentration levels of VCX were identical following i.v. or i.m. injection of PCX, the described difference could be overlooked. Therefore, in the present study, the i.v. route was selected to investigate the pharmacokinetic profile of PCX.

## Conclusions

An efficient UPLC-MS/MS method for the simultaneous determination and quantification of PCX, VCX, and IS from rat plasma was developed. The detection was performed on a TQD in MRM mode using positive ESI. The method was validated to meet the requirements for the pharmacokinetic studies of PCX in rat plasma and could be applied to assess the pharmacokinetic profile of human volunteers in future studies.

## Data Availability

The datasets used and/or analyzed during the current study are available from the corresponding author on reasonable request.

## Abbreviations

PCX: Parecoxib
COX-2: Cyclooxygenase 2
i.v.: Intravenous
i.m.: Intramuscular
VCX: Valdecoxib
CYP3A4: Cytochrome P450 3A4
CYP2C9: Cytochrome P450 2C9
UPLC-MS/MS: Ultra-performance liquid chromatography-tandem mass spectrometry
QSM: Quaternary Solvent Manager
SM-FTN: Sample Manager with Flow-Through Needle
ESI: Electrospray ionization
MRM: Multiple reaction monitoring
QC: Quality control
IS: Internal standard
LLOQ: Lower limit of quantitation
RE: Relative error
RSD: Relative standard deviations
DAS: Drug and Statistics
LLE: Liquid-liquid extraction
SPE: Solid phase extraction
AUC: Area under the plasma concentration-time curve
C_max_: Peak plasma concentration
